# P-1270. Using Machine Learning and ICD-10 Codes to Identify Cases of Laboratory-Confirmed Influenza, 2015-2020, US Flu VE Network

**DOI:** 10.1093/ofid/ofae631.1451

**Published:** 2025-01-29

**Authors:** Haris M Ahmad, Lauren Beacham, Manjusha Gaglani, Kempapura Murthy, Huong Nguyen, Edward Belongia, Mary Patricia Nowalk, Krissy Moehling, Erika L Kiniry, Rachael P Doud, Emily T Martin, Arnold Monto, Nathaniel Lewis, Sascha Ellington, Samantha M Olson, Jessie R Chung, Brendan Flannery

**Affiliations:** Centers for Disease Control and Prevention, Atlanta, Georgia; Centers for Disease Control and Prevention, Atlanta, Georgia; Baylor Scott & White Health, Temple, TX; Baylor Scott and White Health, Temple, Texas; Marshfield Clinic Research Institute, Marshfield, Wisconsin; Marshfield Clinic Research Institute, Marshfield, Wisconsin; University of Pittsburgh, Pittsburgh, PA; University of Pittsburgh, Pittsburgh, PA; Kaiser Permanente Washington Health Research Institute, Seattle, Washington; KPWHRI, Lake Stevens, Washington; University of Michigan, Ann Arbor, Michigan; University of Michigan, Ann Arbor, Michigan; Centers for Disease Control and Prevention, Atlanta, Georgia; CDC, Atlanta, Georgia; Centers for Disease Control and Prevention, Atlanta, Georgia; Centers for Disease Control and Prevention, Atlanta, Georgia; Centers for Disease Control and Prevention, Atlanta, Georgia

## Abstract

**Background:**

The International Classification of Diseases (ICD-10) has unique codes for influenza. Accurate classification of influenza is necessary to calculate unbiased vaccine effectiveness and typically requires laboratory testing. We used the US Influenza Vaccine Effectiveness (Flu VE) Network to determine if outpatient influenza-positive cases and influenza-negative controls could be identified by the presence or absence of certain codes to better understand the role of ICD-10 in estimating VE.

**Table:**
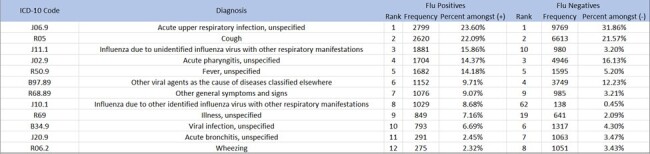
Twelve most common ICD-10 codes among influenza-positive outpatients in the US Flu VE Network and their corresponding rankings and frequencies among influenza-negative patients, 2015-16 through 2019-20 influenza seasons

**Methods:**

During the 2015-16 through 2019-20 influenza seasons, 5 US Flu VE sites enrolled outpatients aged ≥6 months with medically attended acute respiratory illness (MAARI) defined as self-reported new or worsening cough lasting ≤7 days and tested patients for influenza by RT-PCR. We determined the frequencies of ICD-10 codes among influenza cases and controls. We also used classification and regression trees (CART) models to evaluate ICD-10 codes as predictors of influenza test results.

**Figure:**
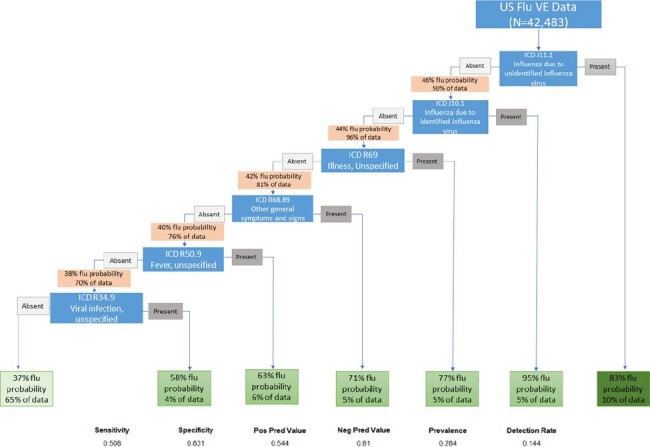
Classification and Regression Tree using ICD-10 codes to determine influenza testing status for outpatients in the US Flu VE Network, 2015-16 through 2019-20 influenza seasons

**Results:**

Over 5 seasons in the US Flu VE, we identified 2,711 ICD-10 codes assigned to enrollments among MAARI patients. Among 11,861 patients with laboratory-confirmed influenza, we identified a total of 1,234 unique ICD-10 codes versus 2,450 ICD-10 codes among 30,622 patients who tested negative for influenza. Codes for cases and controls overlapped substantially, with Diseases of the Respiratory system (J00-J99) being the most frequent (78%) among both. Among cases and controls, J06.9 (Acute upper respiratory infection, unspecified) and R05 (Cough) were the most frequent (Table). CART analysis identified a subset of 6 optimal MAARI-associated ICD-10 codes that identified 6,112 (52%) influenza cases, and these codes were absent for 25,518 (83%) test-negative controls totaling 31,630 (74%) cases and controls (Figure A).

**Conclusion:**

Our findings show the importance of laboratory-confirmed influenza outcomes for influenza VE analyses; machine learning may be useful in identifying MAARI-associated ICD-10 diagnostic codes to define patients for inclusion in test-negative VE studies. In our study, we identified a restricted list of ICD-10 codes that could be used to improve VE estimates in electronic medical record systems with access to testing data.

**Disclosures:**

**Huong Nguyen, PhD, MPH**, CSL Seqirus: Advisor/Consultant|CSL Seqirus: Grant/Research Support|GSK: Grant/Research Support|ModernaTX: Advisor/Consultant|ModernaTX: Grant/Research Support **Mary Patricia Nowalk, PhD**, AstraZeneca - Icosavax: Grant/Research Support|GSK: Advisor/Consultant|Merck & Co.: Grant/Research Support|Sanofi: Grant/Research Support **Arnold Monto, MD**, Roche: Advisor/Consultant

